# Continuous local intravenous heparin infusion after re‐exploration for venous congestion in free‐flap breast reconstruction: A case series

**DOI:** 10.1002/ccr3.6858

**Published:** 2023-03-19

**Authors:** Kazuyuki Kubo, Miho Kirita, Atsumori Hamahata, Hiroyuki Sakurai

**Affiliations:** ^1^ Division of Breast Surgery Saitama Cancer Center Saitama Japan; ^2^ Division of Plastic and Reconstructive Surgery Saitama Cancer Center Saitama Japan; ^3^ Department of Plastic and Reconstructive Surgery Tokyo Women's Medical University Tokyo Japan

**Keywords:** breast reconstruction, flap salvage, free flap, heparin infusion, venous congestion

## Abstract

We demonstrated local intravenous heparin infusion to salvage flaps after re‐exploration for postoperative venous congestion after free‐flap breast reconstruction. All flaps were salvaged using local intravenous heparin infusion without major complications. Local intravenous heparin infusion is an effective and safe procedure.

## Graphical abstract

Local intravenous heparin infusion is an effective and safe procedure to salvage flaps after re‐exploration for postoperative venous congestion of free‐flap breast reconstruction.

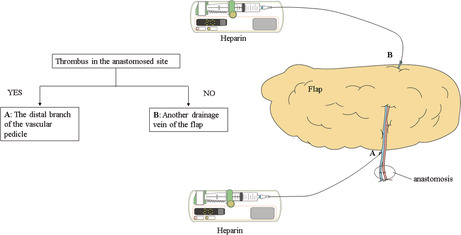

## 1 INTRODUCTION

As free‐flap procedures including microsurgical techniques advance, various free flaps have been used for breast reconstruction.^1^ However, free‐flap reconstruction carries the potential risk for circulatory compromise, which could lead to flap loss. Postoperative venous congestion is observed more frequently than arterial compromise and can be caused by various factors, such as thrombus in the anastomosed site, flap(s) compression, or kinking of the vascular pedicle. Although early detection and re‐exploration are essential for salvaging flap congestion, flap loss after re‐exploration is not rare.

Various methods to improve salvage rates after re‐exploration for venous congestion have been described.^2^ Among these, the administration of anticoagulants or thrombolytic agents is considered to be an effective method.^3^, ^4^, ^5^ We demonstrated local intravenous heparin infusion to salvage flaps after re‐exploration for postoperative venous congestion after free‐flap breast reconstruction. We also describe the details of the catheterization technique, heparin infusion methods, and patient data.

## 2 METHODS

The present study was approved by the Ethics Committee of Saitama Cancer Center (Saitama, Japan). Data from the medical records of six patients, who underwent re‐exploration due to postoperative venous congestion and postoperative local intravenous heparin infusion at the Saitama Cancer Center between January 2013 and December 2021, were retrospectively reviewed. Operative data were also collected, including those on type of flap, flap salvage, site of venous catheterization, heparin dose, cause(s) of flap congestion, re‐anastomosed veins, and postoperative complications.

### 2.1 Primary operations

The types of flaps were selected among the abdominal‐based free (ABF) flap or profunda artery perforator (PAP) flap, according to the volume of the contralateral breast mount. The vascular pedicles of the ABF flaps were the deep inferior epigastric vessels. These PAP flaps are perforators of the profunda femoris vessels. The internal mammary artery and vein were selected as recipient vessels. Venous and arterial anastomoses were performed using the end‐to‐end technique. At the conclusion of the primary operations, the authors confirmed that there were no signs of circulatory compromise by checking flap(s) color, performing pin prick tests, and investigating sounds emitted by Doppler stethoscopes. None of the patients underwent postoperative systemic anticoagulation therapy.

### 2.2 Re‐exploration for venous congestion

Re‐exploration was initiated as soon as possible after the recognition of flap congestion, and was performed with the patient under general anesthesia. After opening the wound and removing the hematoma, the vascular anastomosed site and the vascular pedicle were assessed to determine the cause of congestion. If a thrombus was detected in the anastomosed site, thrombus removal and re‐anastomosis were performed. A vein graft was applied in case of vessel shortage. In all re‐anastomosis cases, the internal mammary artery and vein were chosen as the recipient vessels. If the pedicle was kinked, the vascular position was straightened. If the pressure of the flap was too high, the flap was trimmed and an artificial dermis was used to decrease flap pressure. After blood circulation in the flap was improved, intraoperative venous catheterization was performed as described below.

### 2.3 Intraoperative venous catheterization

**FIGURE 1:**
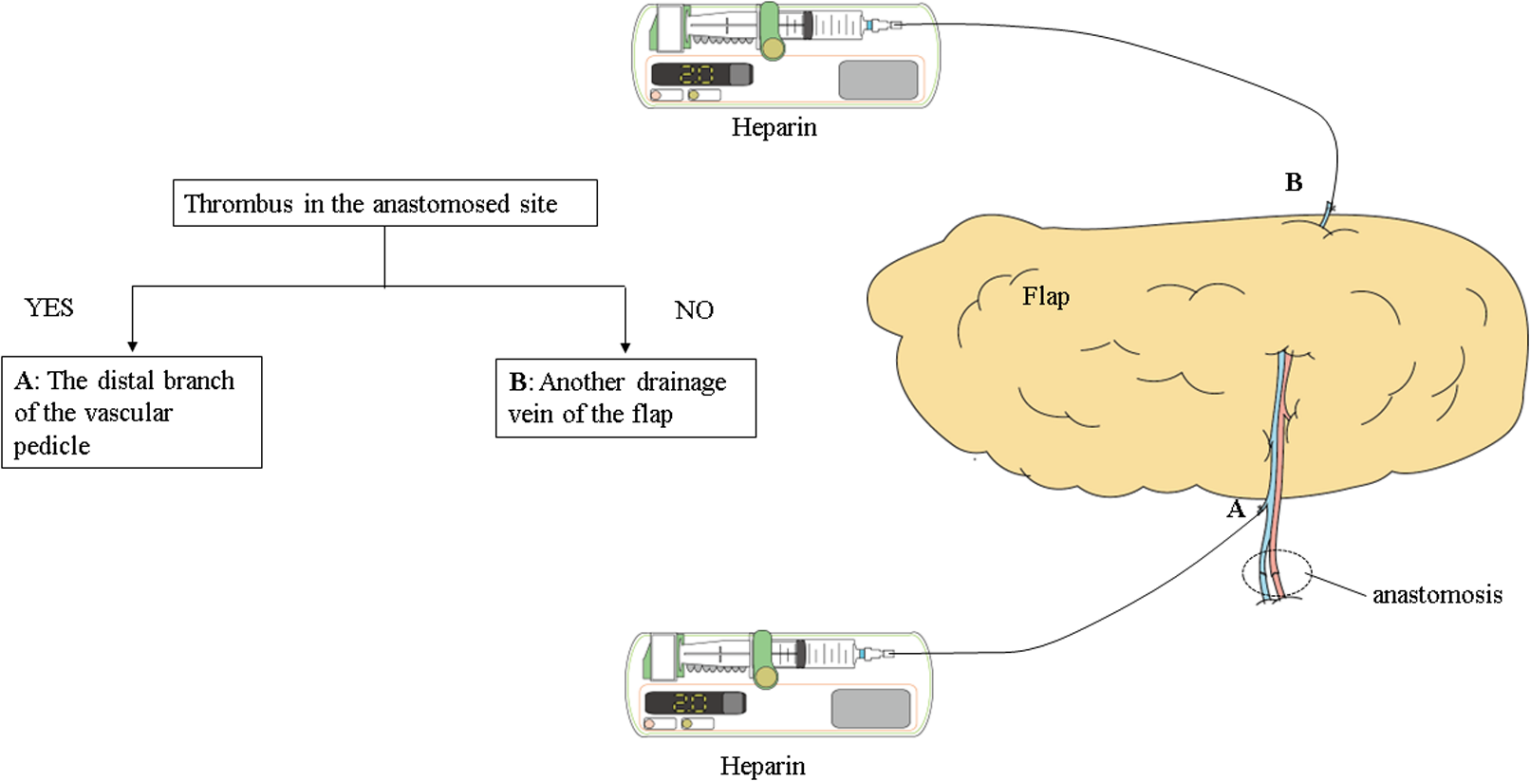
The selection of a catheterized vein with or without a thrombus in the venous anastomosed site. If a thrombus was detected in the venous anastomosed site, the catheter was inserted into the distal branch of the vascular pedicle (A). If a thrombus was not detected in the anastomosed site, the catheter was inserted into another drainage vein of the flap (B).

After blood circulation in the flap was improved, an epidural catheter (0.85 mm outer diameter, B Braun) was inserted into the flap vein. If a thrombus was detected in the venous anastomosed site, the catheter was inserted into the distal branch of the vascular pedicle. If a thrombus was not detected in the anastomosed site, the catheter was inserted into another drainage vein of the flap. Another drainage vein was the superficial inferior epigastric vein of the ABF flap and accessory saphenous vein of the PAP flap (Figure 1).

**FIGURE 2:**
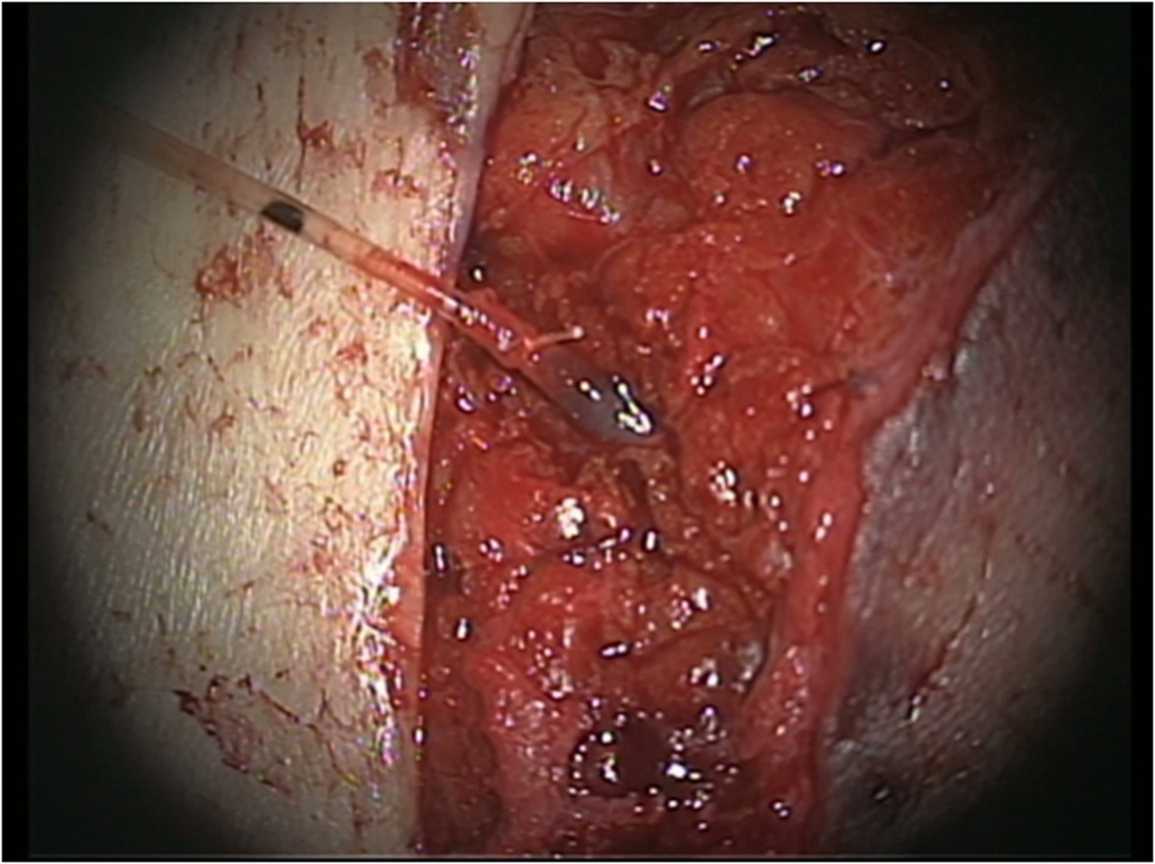
The catheter was inserted into the flap vein and ligated using a 4–0 silk suture.

Venous catheterization was performed using a surgical microscope. The catheter was inserted 2–3 cm proximal to the vein. After catheter insertion, the vein was ligated using a 4–0 silk suture (Figure 2). Manual saline insertion was repeated until the saline flowed out of the edges of the flaps, which was considered to be a sign of saline perfusion of the flap. Subsequently, the catheter was led through the skin and sutured to several places to form a loop.

Continuous heparin infusion was initiated through the catheter postoperatively. The solution contained 3000 U of unfractionated heparin (3 ml) and 45 ml of normal saline. The solution was injected at a rate of 2 ml/h (125 U/h) using syringe pumps. On postoperative day 5, heparin infusion was discontinued, and the catheter was removed the next day. Manual compression was performed for several minutes after catheter removal to avoid bleeding and to preclude hematoma formation.

## 3 RESULTS

A summary of the re‐exploration cases is presented in Table 1. All flaps were salvaged using local intravenous heparin infusion. In three cases, the distal branch of the pedicle veins was selected as the insertion vein. These three cases, all included ABF flaps, and the pedicle veins were the deep inferior epigastric veins. In the other three cases, another flap drainage vein was selected as the insertion vein (superficial inferior epigastric veins of ABF flaps [*n* = 2] and accessory saphenous vein of PAP flap [*n* = 1]). The heparin dose range was 2.1–2.6 U/kg/h. Venous thrombi were detected in three patients; the cause of congestion, except for venous thrombus, was compression of the flap (*n* = 2) and kinking of the pedicle (*n* = 1). Postoperative complications were observed in one case, with breast skin necrosis observed in case 6, which required a secondary skin graft. No postoperative complications, such as hematoma or bleeding, were observed after catheter placement and removal.

**TABLE 1:** Summary of cases

No	Type of flap	Flap salvage	Site of venous catheterization	Heparin dose (U/h)	Cause of flap congestion	Re‐anastomosed vein	Postoperative complications
1	ABF	○	Pedicle (DIEV)	2.1	Thrombus	○	–
2	ABF	○	Pedicle (DIEV)	2.2	Thrombus	○	–
3	PAP	○	ASV	2.6	Kinking		–
4	ABF	○	Pedicle (DIEV)	2.1	Thrombus	○	–
5	ABF	○	SIEV	2.5	Compression		–
6	ABF	○	SIEV	2.3	Compression		Skin necrosis

Abbreviations: ABF, abdominal‐based free‐flap; ASV, accessory saphenous vein; DIEV, deep inferior epigastric vein; PAP, profunda femoris artery perforator flap; SIEV, superficial inferior epigastric vein.

## 4 CASE PRESENTATION (CASE 1)

**FIGURE 3:**
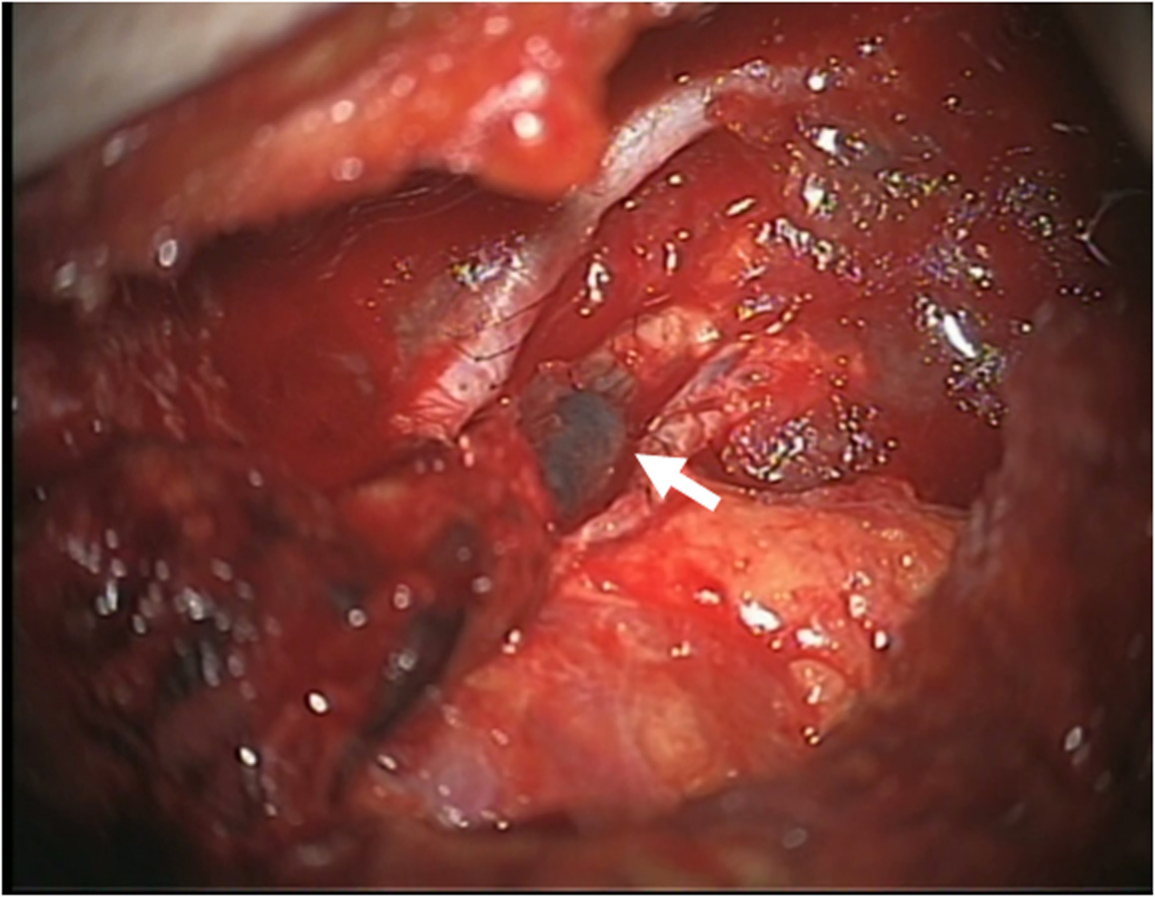
A thrombus was detected in the venous anastomosed site (arrow).

A 46‐year‐old woman underwent immediate abdominal‐based free flap breast reconstruction for the left breast cancer. Right deep inferior epigastric artery and vein were anastomosed to left internal mammary artery and vein. Blood circulation of the flap was stable after surgery, however, the flap color rapidly changed to congestive color at postoperative day 5. Re‐operation was performed immediately. A thrombus was detected in the venous anastomosed site (Figure 3). After removal of the thrombus and re‐anastomosis of the vein, blood circulation improved.

**FIGURE 4:**
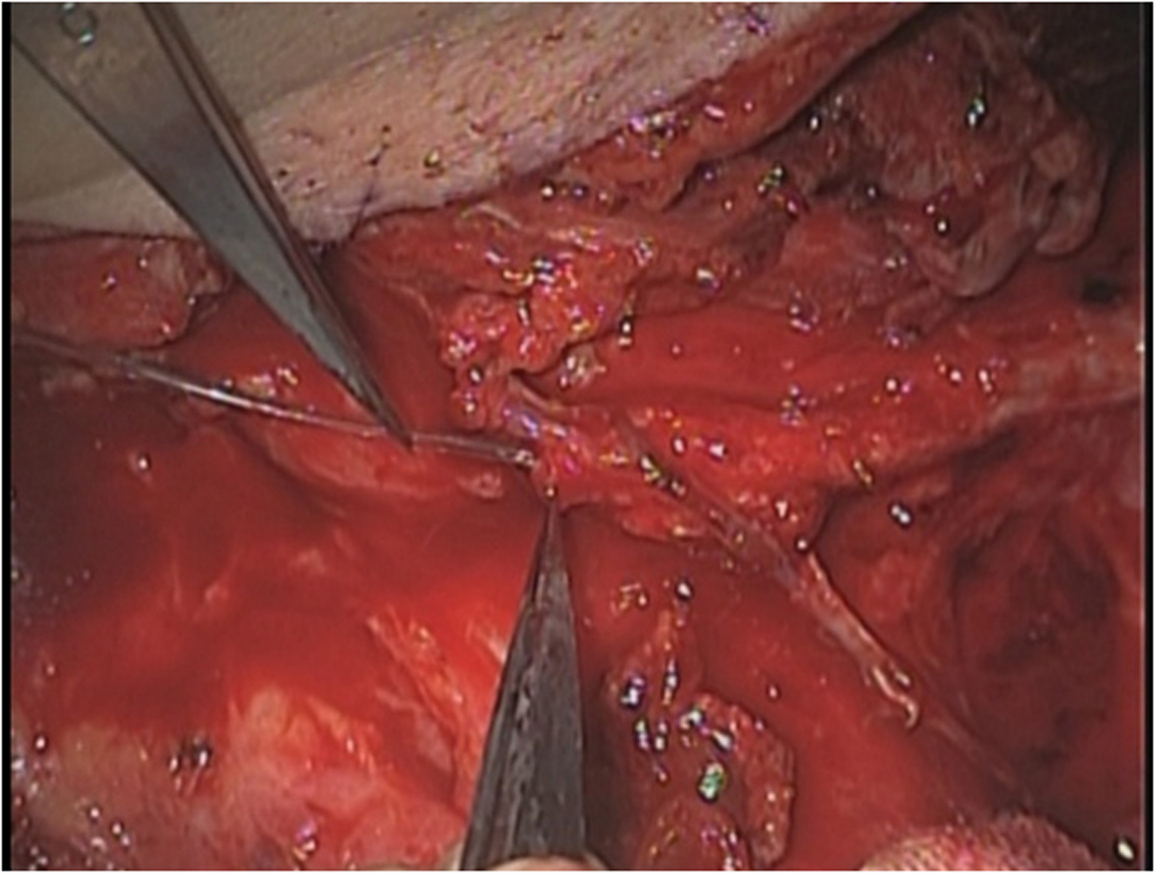
The catheter was inserted to the distal branch of the pedicle vein.

**FIGURE 5:**
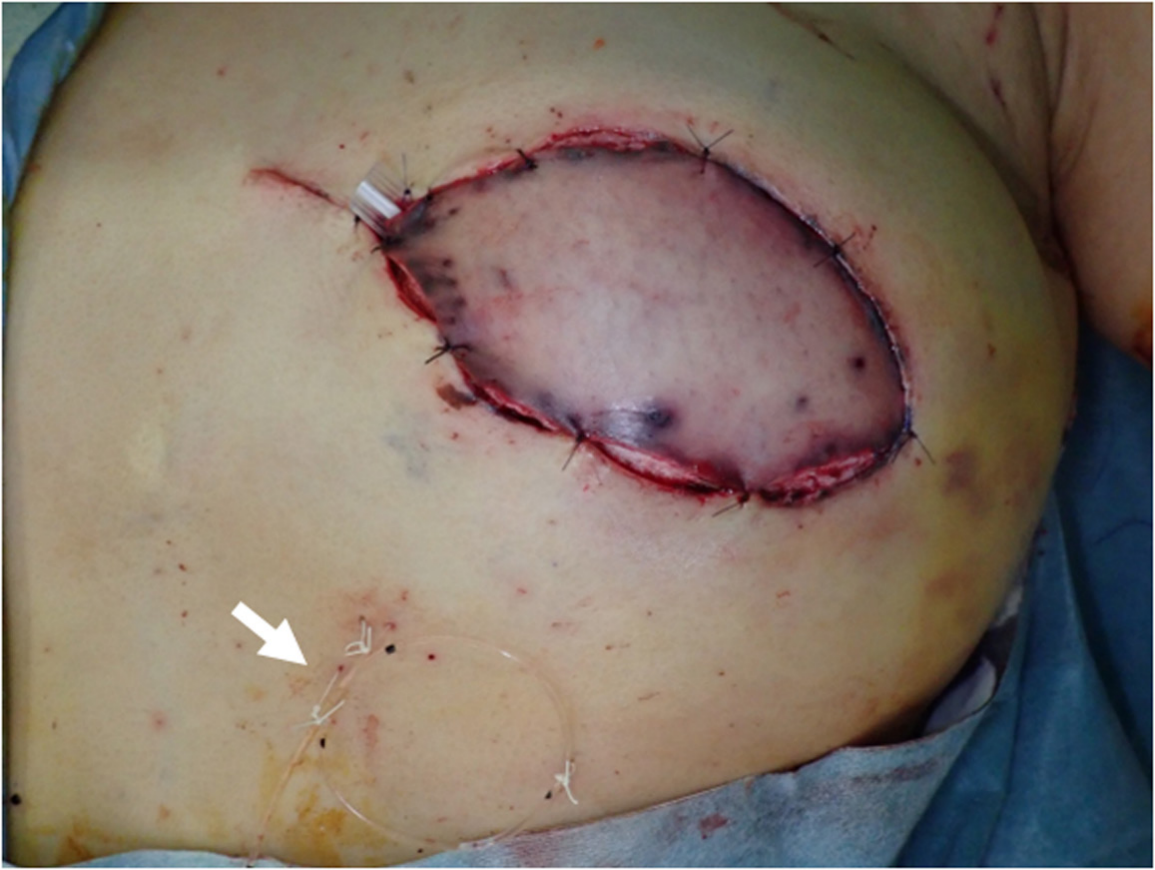
The flap color remained congestive just after surgery. The catheter was placed (arrow).

**FIGURE 6:**
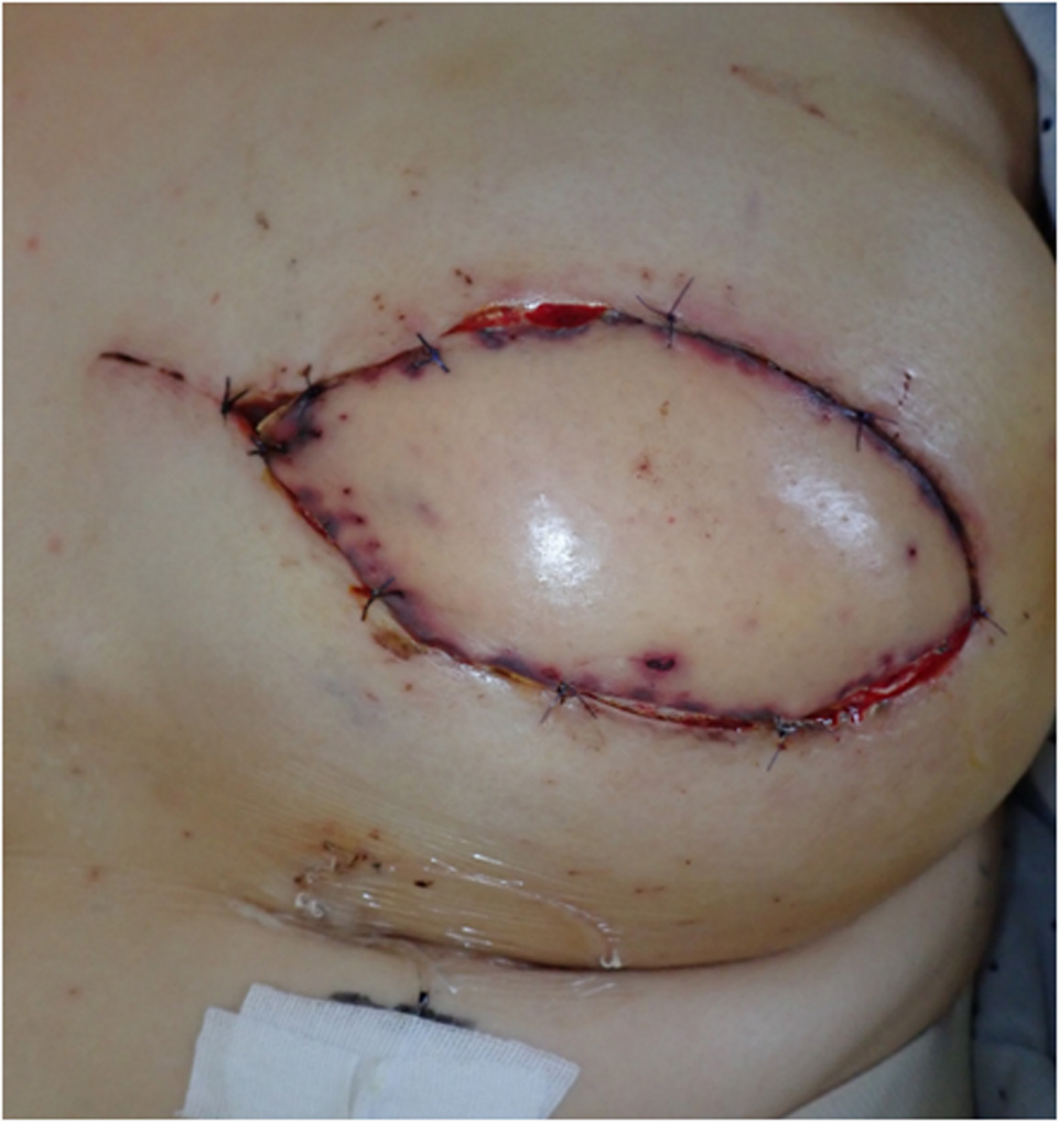
The flap color turned into normal 5 days after re‐operation.

After that, intravenous catheterization and continuous heparin infusion were performed. The catheter was inserted to the distal branch of the pedicle vein (Figure 4). Although the flap color remained congestive just after surgery (Figure 5), it gradually turned into normal over time (Figure 6). The flap survived completely and there was no postoperative complication.

## 5 DISCUSSION

Venous congestion is classified as either intraoperative or postoperative according to the onset of venous outflow insufficiency.^6^ Postoperative venous congestion can develop owing to a thrombus in an anastomosed site or non‐thrombotic causes such as kinking of the pedicle or over‐compression. On the other hand, except for technical failures, such as perforator injury or anastomosis errors, the primary cause of intraoperative flap congestion is anatomical factors, specifically the existence of another venous drainage system.^7^ If there is another dominant drainage vein, flap congestion will occur intraoperatively, even in the absence of complications of venous anastomosis. In such cases, “super‐drainage” is necessary to avoid venous congestion.^6^, ^7^ In the current study, intraoperative blood circulation was normal in all re‐exploration cases at the conclusion of the primary operation. Therefore, pedicle veins were believed to be dominant for flap circulation, and other drainage veins (superficial inferior epigastric vein and accessory saphenous vein) were candidates for catheterized veins, not for donors of super‐drainage veins.

Although various methods to improve salvage rates for venous congestion have been reported, the salvage rate after re‐exploration for circulatory compromise has been reported to be 44%–80%.^8^ Administration of anticoagulants or thrombolytic agents after re‐exploration is considered to be effective^3^, ^4^, ^5^; however, the best agent and administration route remain unclear because no prospective comparative studies have been performed. We described local continuous heparin infusion through a catheter inserted into the flap vein and the effectiveness of this method through a discussion of the pharmacological action, administration route, and heparin dose.

It is well‐known that heparin has anticoagulant activity. It combines with antithrombin‐III and inhibits blood coagulation factor X and the conversion of prothrombin to thrombin.^9^ These pharmacological actions prevent microthrombus formation and increase flap blood flow.^10^, ^11^, ^12^ In addition, some studies have suggested that heparin may have a protective effect against reperfusion injury in the microvascular endothelium, which may cause new thrombus formation.^11^ Unfractionated heparin has an antagonistic drug (protamine) and can be monitored by measuring activated partial thromboplastin time (aPTT)^9^; therefore, an appropriate dose of heparin can be adjusted, and it is safe for continuous administration. Based on these merits, heparin has been selected as a therapeutic agent for the compromise of flap circulation in many previous studies and in the current investigation.

Systemic and local administration routes for heparin infusion have been reported after re‐exploration for venous congestion of free flaps.^3^, ^10^, ^13^ Systemic administration is not technically difficult because it does not require an injection route into the flap vessels. Systemic heparin administration using insufficient doses, however, does not benefit flap salvage.^11^ To prolong aPTT to the effective range (1.5–2.3 times higher than normal), a high dose of systemic heparin (>18 U/kg/h) is required.^13^ This high dose causes bleeding and the formation of hematoma(s).^13^ In contrast, local administration does not require a high dose, because in this case, heparin acts directly at a high concentration in the flap.^10^, ^11^ Local administration routes are divided into two types: intra‐arterial and intravenous. Local intra‐arterial heparin administration requires catheter access to the flap artery or the recipient artery and carries a risk for bleeding and hematoma formation during catheter placement, which in turn may cause arterial compromise. In addition, a second surgery is sometimes required to remove the catheter.^10^ Several previous studies have showed the efficacy of local intravenous heparin injection(s), similar to the current study.^8^, ^10^, ^12^ The local intravenous route is safer than the systemic and local intra‐arterial routes. It does not require a high dose of heparin and has a lower risk for catheter‐related complications, such as bleeding and hematoma formation. Matsumine reported a venous pressure measuring system using catheters inserted into the flap vein to monitor flap circulatory compromise.^14^ The author reported no catheter‐related complications among 271 cases with venous catheterization. Similarly, catheter‐related complications were not observed in the present study.

Although local heparin administration does not require a high dose, the optimal dose remains unclear. Animal experiments have revealed that even low‐dose heparin (5–6 U/kg/h) delivered locally to the flap has sufficient and beneficial action.^11^ Another in vivo study showed that the rate of postoperative bleeding complications was significantly higher in cases of local administration of heparin at >6.5 U/kg/h.^13^ We decided on the dose of heparin—not only in reference to these reports—but also considering the condition of re‐explorations. Re‐exploration of flap circulatory compromise is always an emergency procedure. In such abnormal conditions, detailed adjustment of the heparin dose may cause medical errors. Therefore, the dose was set to 3000 U/24 h in simple order, and the dose fell within the rage of 2.0–3.0 U/kg/h as a result.

The vein in which the catheter was inserted was selected as described above. In cases with a thrombus in the venous anastomosed site, the distal branch of the vascular pedicle was selected as the vein to catheterize. In cases without thrombus in the anastomosed site, another drainage vein was selected. This choice was based on the primary target of heparin to prevent the relapse of thrombus in the anastomosed site or microthrombus in the entire flap vein.

Vascular ingrowth can be established 5–7 days after flap transplantation.^15^ Based on this assumption and considering the risk for catheter‐related complications that may develop with long‐term catheter placement, such as catheter fever, 5 days were set as the duration of continuous heparin infusion in the current study. After interrupting heparin infusion, the catheter was inserted for 24 h in preparation of restarting the infusion owing to relapse of venous congestion.

## 6 CONCLUSION

Local intravenous heparin infusion is an effective and safe procedure to salvage flaps after re‐exploration for postoperative venous congestion.

## AUTHOR CONTRIBUTIONS

KK drafted and prepared the original manuscript. MK and AH obtained and analyzed the patient data. AH and HS contributed to the conception and design of the study, and revised the manuscript. All authors approved of the final submission and have made significant contributions to the manuscript.

## FUNDING INFORMATION

None.

## CONFLICT OF INTEREST

The authors declare there is no conflict of interest.

## ETHICAL APPROVAL

The present study was approved by the Ethics Committee of Saitama Cancer Center (Saitama, Japan).

## CONSENT

Written informed consent was obtained from the patient to publish this report in accordance with the journal's patient consent policy.

## Data Availability

The data that support the findings of this study are available from the corresponding author upon reasonable request.
